# Efficacy of Carbohydrate Ingestion on CrossFit Exercise Performance

**DOI:** 10.3390/sports5030061

**Published:** 2017-08-14

**Authors:** Jaden A. Rountree, Ben M. Krings, Timothy J. Peterson, Adam G. Thigpen, Matthew J. McAllister, Megan E. Holmes, JohnEric W. Smith

**Affiliations:** 1 Department of Kinesiology, University of Alabama, Tuscaloosa, AL 34587, USA; 2 Department of Kinesiology, Mississippi State University, Starkville, MS 39762, USA; bmk216@msstate.edu (B.M.K.); tjp91@msstate.edu (T.J.P.); agt42@msstate.edu (A.G.T.); mjm639@msstate.edu (M.J.M.); mholmes@colled.msstate.edu (M.E.H.); jws597@msstate.edu (J.W.S.)

**Keywords:** high-intensity exercise, resistance training, ergogenic aids, strength

## Abstract

The efficacy of carbohydrate (CHO) ingestion during high-intensity strength and conditioning type exercise has yield mixed results. However, little is known about shorter duration high-intensity exercise such as CrossFit. The purpose of this study was to investigate the performance impact of CHO ingestion during high-intensity exercise sessions lasting approximately 30 min. Eight healthy males participated in a total of four trials; two familiarizations, a CHO trial, and a similarly flavored, non-caloric placebo (PLA) trial. CrossFit’s “Fight Gone Bad Five” (FGBF) workout of the day was the exercise model which incorporated five rounds of maximal repetition exercises, wall throw, box jump, sumo deadlift high pull, push press, and rowing, followed by one minute of rest. Total repetitions and calories expended were summated from each round to quantify total work (FGBF score). No difference was found for the total work between CHO (321 ± 51) or PLA (314 ± 52) trials (*p* = 0.38). There were also no main effects (*p* > 0.05) for treatment comparing exercise performance across rounds. Based on the findings of this study, it does not appear that ingestion of CHO during short duration, high-intensity CrossFit exercise will provide a beneficial performance effect.

## 1. Introduction

The ergogenic effects of carbohydrate (CHO) ingestion during continuous, prolonged aerobic exercise has been well documented [1,2]. However, the benefits of CHO ingestion during resistance exercise has not been as consistent. Recent research has reported the beneficial effects of CHO ingestion on shorter duration exercise (<25 min) [3], intermittent exercise (>60 min) [4,5,6], and strength and conditioning exercise (>70 min) [7]. The increased popularity of high-intensity exercise methods, such as CrossFit (CF), indicates that such methods are increasingly common among athletes seeking to improve training and performance. However, there is a significant lack of randomized controlled trials involving CF specific-based exercise and supplementation of ergogenic aids, specifically CHO.

CrossFit day-to-day workouts have a great deal of variance based on the workout of the day. Babiash et al. [8] found that two common CF workouts resulted in heart rates averaging ~90% of heart rate maximum and aerobic intensities averaging ~85% maximal oxygen consumption (VO_2max_). It is not uncommon for CF workout tasks to exceed VO_2max_ intensities for brief but repeated time periods. Muscle glycogen levels have been shown to decline significantly in as little as 20 min with exercise intensities of 120% VO_2max_ [9]. Muscle glycogen decline has also been shown to occur at an accelerated rate following prolonged and high-intensity resistance training [10,11,12]. These declines may result in diminished performance as glycogen stores becomes less available. CHO ingestion during a CF session could potentially reduce the rate of glycogen depletion [13,14], as well as provide an additional fuel source to maintain or improve performance.

Previous investigations have shown CHO supplementation during resistance exercise sessions to be beneficial for performance [15,16,17,18]. In an investigation examining the effects of CHO ingestion during a strength and conditioning exercise session, CHO was found to be more beneficial than non-CHO supplementation, when ingesting CHO at rates of 15–30 g/h [7]. However, CHO ingestion has also been shown to not enhance resistance exercise performance [19]. With minimal research regarding CHO supplementation in CF performance, it is difficult to apply the methodologies employed in previous resistance exercise research to CF. Additionally, it is suggested that the CHO mouth rinse technique be utilized during short-duration high-intensity exercise lasting <45 min [20] to see an ergogenic effect from CHO. The CHO mouth rinse technique has been shown to be beneficial during shorter duration high-intensity exercise [21]. Beneficial effects may be due to the enhancement of areas of the brain associated with reward and motor control [22], and motor performance and sensory perception [23]. Due to the findings of Carter et al. [24], who observed no-performance benefits with infusion of CHO during a 1-h time trial, Carter et al. [25] suggested that the performance benefits of CHO ingestion during exercise (~60 min), seen by the same lab [26,27], may be explained via mouth rinse mechanisms. Therefore, a potential performance enhancement from the ingestion of exogenous CHO during CF exercise may be explained by the independent or combined effects of oral sensing in the mouth and exogenous CHO being readily available for substrate utilization in the muscle.

Due to the increasing prominence of CF, methods to improve performance are likely being sought. Building upon the knowledge from activities that are contributors to common CF workouts, the purpose of the present study was to investigate the influence of CHO ingestion on performance during a Fight Gone Bad Five (FGBF) CF workout. It was hypothesized that CHO supplementation would improve FGBF performance.

## 2. Methods

### 2.1. Participants

Eight healthy, college-aged, CF-trained males (age: 22 ± 1.8 years, height: 177 ± 7 cm, and body mass: 81.3 ± 7.2 kg) participated in the study. Participants had to be resistance training for at least the past six months, as well as CF specific training for the previous three months. Prior to participating in the study, participants were informed of all potential risks and benefits and gave written informed consent per the University’s Institutional Review Board. Participants were allowed water within the 10–12 h preceding the exercise intervention to aid and maintain adequate hydration status. Additionally, all participants abstained from consuming any additional supplementation, including caffeine, throughout the entirety of the study and refrained from all exercise in the 48 h prior to trials.

### 2.2. Experimental Design

Participants reported to the University Recreational Center’s training facility following a 10–12 h overnight fast. Each participant completed four trials separated by at least seven days. The first two trials served as familiarization trials to allow any learning effects of FGBF to stabilize [28]. The first trial required no restriction on fluid type or intake volume during the session. During the second trial, each participant was restricted to a 6% CHO beverage containing 41.6 mL of water provided immediately prior to the warm-up, prior to the initiation of the FGBF exercises, and during the one-minute rest breaks within FGBF (six total ingestions). This schedule and the intake volumes were maintained for the third and fourth trials. A CHO or placebo (PLA) beverage was used in the third and fourth trials using a double blind, randomized cross-over design. Participants were also instructed to maintain normal dietary patterns three days prior to each trial.

### 2.3. Experimental Protocol

#### 2.3.1. Anthropometrics and Hydration Status

Participants’ mass (Ironman Innerscan, Tanita, Arlington, IL, USA) and hydration status was assessed upon arrival to the facility. Hydration was assessed using urine refractometry (PAL-10S Pocket Refractometer, ATAGO, Tokyo, Japan). One participant arrived with a urine specific gravity greater than 1.025, considered hypohydrated, and was rescheduled for another day. For each trial, pre- and post-nude body mass were recorded. 

#### 2.3.2. Experimental Trials

Prior to beginning the exercise intervention, participants performed a standardized dynamic warm-up. The dynamic warm-up consisted of four exercises: rowing, forward lunges with arm circles, 4.5 kg wall-ball throws, and bear crawls. The warm-up lasted ~5 min. The exercise intervention was the “Fight Gone Bad Five” (FGBF) benchmark workout. FGBF consists of five rounds of wall throw, box jump, sumo deadlift high pull, push press, and rowing ergometry. Exercise order, resistances, and box height are listed in Table 1. Participants were instructed to complete as many repetitions as possible at each station for one minute prior to moving to the next station. Immediately upon finishing one exercise, the timer was started for the next exercise. Each repetition for wall throw, sumo deadlift high pull, box jump, and push press were assessed for successful completion by a Level 1 CF trainer. The same CF trainer was used for the entirety of the study for consistency. To quantify performance, one point was given for each repetition during wall throws, sumo deadlift high pull, box jump, and push press. Repetitions were only counted if participants completed the exercise through a full range of motion and with proper form. Incorrectly performed repetitions were not noted during experimental trials. Rowing performance was scored as caloric expenditure (kcal) for work completed in one minute on a rower (Model D, Concept2 Inc., Morrisville, VT, USA) at a resistance of eight. After completing each of the five stations, participants had one minute of rest before beginning the next round. The exercise task lasted 30 min. Only vital research personnel were allowed in the exercise room and no music was permitted to reduce influence from external factors.

#### 2.3.3. Supplementation

CHO and PLA beverages were provided by Sqwincher (The Sqwincher Corporation, Columbus, MS, USA). The CHO supplement was a 6% sucrose/dextrose solution, both rapidly oxidized carbohydrates [29]. Participants received 16 grams of CHO in ~250 mL of fluid over 30 minutes during the supplementation trial. The PLA was a non-caloric beverage (Sqwincher Zero) containing sucralose and aspartame to mimic the sweetness of the CHO solution. Beverages containing a 6% CHO solution in 41.6 mL of water were provided immediately prior to the warm-up, prior to the initiation of round one of FGBF, and during the one-minute rest breaks after rounds 1–4 for a total of six ingestions. Beverages were provided in opaque bottles and CHO and PLA supplements had the same amount of sodium and potassium and were the same flavor (fruit punch). All ingestions were monitored by the principal investigator to ensure participant compliance.

### 2.4. Statistical Analysis

All data are reported as means ± standard deviation. To determine the effect of CHO and PLA ingestion on exercise performance between rounds and treatments, a 5 × 2 (round × treatment) repeated measures analysis of variance was used for each exercise. As appropriate, a Bonferonni post hoc test was performed to further identify differences if significant main effects were found. Repetitions from each exercise and caloric expenditure on the rower was summated across all five rounds of FGBF workout to represent the total work. Student’s paired *t*-test was used to assess differences between total work among treatments. An alpha level of *p* < 0.05 was set as statistically significant and all data analyses were performed using SPSS (Version 23; SPSS Inc., Chicago, IL, USA).

## 3. Results

**Figure 1:**
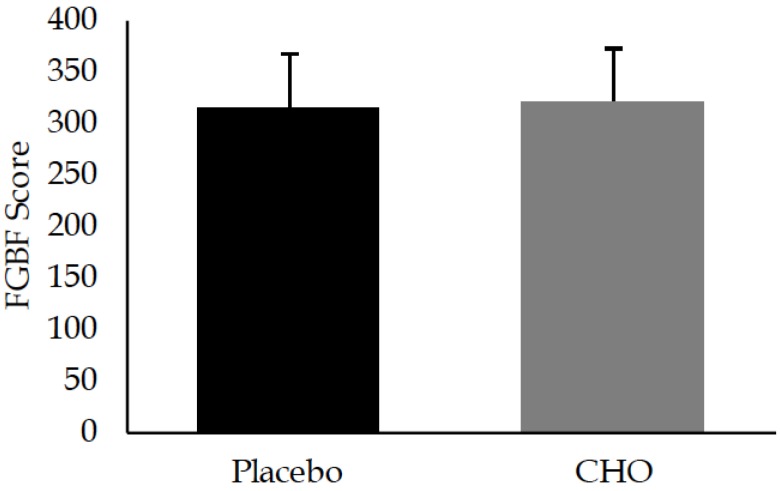
Total scores comparing placebo and carbohydrate during Fight Gone Bad Five. Data are reported as means ± standard deviation.

**Figure 2:**
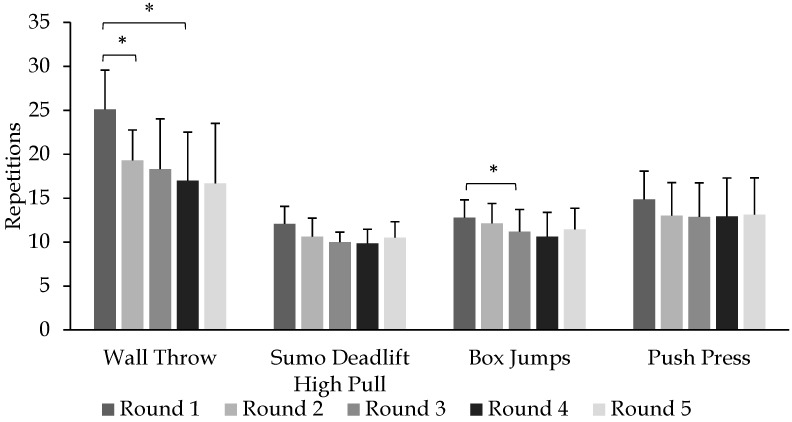
Performance during wall throw, sumo deadlift high pull, box jumps, and push press across five rounds of FGBF. Data are reported as means ± standard deviation. * Statistical significance (*p* < 0.05).

The FGBF scores (determined per CF standards) and mean scores from CHO and PLA trials are displayed in Figure 1. No differences were observed for the total work between CHO and PLA trials (*p* = 0.38). Performance during each exercise and round during both experimental treatments is presented in Table 2. Changes in performance over rounds for wall throw, sumo deadlift high pull, box jumps, and push press are displayed in Figure 2.

Wall throw performance demonstrated no main effect for treatment (*p* = 0.162) but showed performance in rounds to be significantly different (*p* < 0.0001). Wall throw performance in round 1 was significantly greater compared to rounds 2 and 4 (*p* < 0.05), but no differences were observed between rounds 1, 3, and 5 or rounds 2 and 5 (*p* > 0.05). No interactions (round × treatment) were found in wall throw performance (*p* = 0.671).

No main effects were observed for round (*p* = 0.051) or treatment (*p* = 0.776) with sumo deadlift high pull performance. Also, there was no interaction effect (round × treatment) (*p* = 0.631). 

A significant main effect was observed for box jump regarding rounds (*p* = 0.019). Performance during round 1 was significantly greater than performance during round 3 (*p* = 0.024). No treatment main effect (*p* = 0.661) or interaction effect (round × treatment) was observed (*p* = 0.994) for box jumps. 

**Figure 3:**
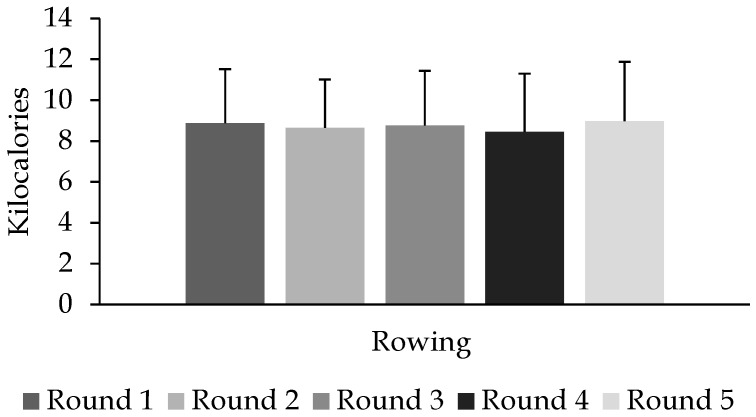
Performance during rowing across five rounds of FGBF. Data are reported as means ± standard deviation.

Performance during push press was not different between treatments (*p* = 0.201), or rounds (*p* = 0.301), and no interaction effect (round × treatment) was observed (*p* = 0.408). Finally, regarding rowing performance, no main effects were observed for round (*p* = 0.812) (Figure 3) or treatment (*p* = 0.409), and no interaction effect was observed (*p* = 0.714).

## 4. Discussion

The aim of the present investigation was to examine the efficacy of CHO supplementation during CF-specific exercise. Our results suggest that acute CHO ingestion does not elicit ergogenic effects during a popular CF workout. Although we did not find treatment effects on performance, wall throw and box jump performance significantly decreased across time and sumo deadlift high pull approached significance (this may have been limited due to the sample size). These round effects suggest that our protocol adequately fatigued subjects. This study adds to the minimal nutritional interventional studies in this relatively new and popular exercise modality. To the authors knowledge, this was also one of the first investigations to assess the efficacy of CHO supplementation during CF.

Previous investigations examining CHO supplementation during high-intensity resistance exercise have found similar results. Kulik et al. [19] examined the effect of CHO supplementation during a high-intensity squat protocol lasting 29 min until failure and found no improvements in performance. During a muscular endurance protocol lasting approximately 39 min, Haff et al. [30] reported glycogen sparing but no significant isokinetic strength differences with CHO ingestion. Non-performance improvements with CHO supplementation may be related to the overall duration, as research supporting the efficacy of CHO ingestion during resistance training performance have employed protocols lasting 77 [15], 71 [7], 57 [16], and 56 min [31]. Recently, Escobar et al. [32] examined the effect of CHO availability through diet, and found that the shorter duration CF workout (lasting 12 min) significantly increased blood lactate levels from rest, and levels remained elevated over the duration of exercise. These results indicate the glycolytic nature of CF exercise due to increased anaerobic metabolism, evident by rapid increases and sustained blood lactate concentrations. A possible mechanism explaining the lack of performance benefit from our study relates to the ability of the gut to empty ingested CHOs, as high-intensity exercise (100% VO_2max_) has been shown to decrease gastric emptying rates [33]. In our study, participants maintained a high-intensity for 5 min followed by 1 min of rest, which may have inhibited the body’s ability to digest the ingested CHO. CF may rely on heavily on CHOs for energy production, but the ability for exogenous CHO to be readily available as a substrate at the muscle may be limited by the high-intensity of exercise and the overall short duration of FGBF.

To simulate CF-specific exercise, the authors utilized a set resistance for each resistance exercise in FGBF. Due to the variation in body mass, overall strength, and muscular endurance of each participant in our study, individual variation may have contributed to our non-significant findings. There is a limited amount of research examining the effects of ergogenic aids on CF performance. In one of the only studies examining CHO supplementation with CF, Outlaw et al. [34] examined the effect CHO in combination with protein on pre-and post-workout supplementation, and found improvements in performance over the duration of six weeks of training with supplementation. Furthermore, Kramer et al., 2016 [35] observed no benefits of CF-specific performance following six days of nitrate supplementation. Due to the limited amount of nutritional intervention research with CF specific performance, more research is needed to establish the efficacy of ergogenic aids to this popular exercise modality.

Our study is not without limitations. First, although our subjects were advised to maintain similar dietary intakes for three days prior to each supplemental intervention, dietary logs were not kept. Therefore, muscle glycogen stores may have varied between trials. Furthermore, we attempted to increase homogeneity by recruiting participants among the same training background, but we are limited by a small sample size (*n* = 8). Future CHO CF exercise studies may benefit by using a standardized load relative to body mass or one repetition max, and by examining CHO supplementation interventions with female CF-trained athletes.

## 5. Conclusions

With a growth in popularity of CF for recreational athletes, more randomized controlled trials examining both physiological responses and nutritional interventions are needed. Due to the competitive nature of CF, it is important to examine the efficacy of any ergogenic aid that may improve performance. Although we did not see any performance improvements, we also did not see a performance decrement. This study was an important step an understanding CHO supplementation during CF-specific exercise.

## Figures and Tables

**Table 1:** Description of Fight Gone Bad Five workout of the day.

Exercise	Intensity
Wall Throws (3.05 m target)	9 kg
Sumo Deadlift High Pull	34 kg
Box Jump	50.8 cm
Push Press	34 kg
Rowing	Max

**Table 2:** Fight Gone Bad Five scores during each round.

Exercise	Round 1	Round 2	Round 3	Round 4	Round 5
**Wall Throw (repetitions)**					
Carbohydrate	26 ± 4	20 ± 4	18 ± 6	18 ± 6	17 ± 8
Placebo	25 ± 5	19 ± 3	19 ± 6	16 ±5	16 ± 6
**Sumo Deadlift High Pull (repetitions)**					
Carbohydrate	12 ± 1	10 ± 1	10 ± 2	10 ± 2	10 ± 4
Placebo	12 ± 3	11 ± 1	10 ± 2	10 ± 2	11 ± 4
**Box Jumps (repetitions)**					
Carbohydrate	13 ± 2	12 ± 2	11 ± 3	11 ± 2	12 ± 3
Placebo	13 ± 2	12 ± 3	11 ± 2	11 ± 3	11 ± 4
**Push Press (repetitions)**					
Carbohydrate	15 ± 3	14 ± 4	13 ± 5	13 ± 3	14 ± 7
Placebo	14 ± 4	13 ± 4	13 ± 3	13 ± 3	12 ± 6
**Row (kilocalories)**					
Carbohydrate	8 ± 3	8 ± 3	9 ± 3	8 ± 3	9 ± 3
Placebo	9 ± 3	9 ± 2	9 ± 3	9 ± 3	9 ± 3

Performance during each round under both experimental conditions. Data are presented as means ± standard deviation.
